# A Curriculum Learning Strategy to Enhance the Accuracy of Classification of Various Lesions in Chest-PA X-ray Screening for Pulmonary Abnormalities

**DOI:** 10.1038/s41598-019-51832-3

**Published:** 2019-10-25

**Authors:** Beomhee Park, Yongwon Cho, Gaeun Lee, Sang Min Lee, Young-Hoon Cho, Eun Sol Lee, Kyung Hee Lee, Joon Beom Seo, Namkug Kim

**Affiliations:** 1 University of Ulsan College of Medicine, Asan Medical Center, Department of Convergence Medicine, Seoul, South Korea; 2 University of Ulsan College of Medicine, Asan Medical Center, Department of Radiology and Research Institute of Radiology, Seoul, Korea; 3 Seoul National University Bundang Hospital, Department of Radiology, Seongnam-si, Gyeonggi-do, Seoul, Korea

**Keywords:** Computer science, Cancer screening, Radiography

## Abstract

We evaluated the efficacy of a curriculum learning strategy using two steps to detect pulmonary abnormalities including nodule[s], consolidation, interstitial opacity, pleural effusion, and pneumothorax with chest-PA X-ray (CXR) images from two centres. CXR images of 6069 healthy subjects and 3417 patients at AMC and 1035 healthy subjects and 4404 patients at SNUBH were obtained. Our approach involved two steps. First, the regional patterns of thoracic abnormalities were identified by initial learning of patch images around abnormal lesions. Second, Resnet-50 was fine-tuned using the entire images. The network was weakly trained and modified to detect various disease patterns. Finally, class activation maps (CAM) were extracted to localise and visualise the abnormal patterns. For average disease, the sensitivity, specificity, and area under the curve (AUC) were 85.4%, 99.8%, and 0.947, respectively, in the AMC dataset and 97.9%, 100.0%, and 0.983, respectively, in the SNUBH dataset. This curriculum learning and weak labelling with high-scale CXR images requires less preparation to train the system and could be easily extended to include various diseases in actual clinical environments. This algorithm performed well for the detection and classification of five disease patterns in CXR images and could be helpful in image interpretation.

## Introduction

Chest posterior-anterior (PA)-X-ray (CXR) is considered one of the most accessible types of radiological examinations to screen for and diagnose pulmonary problems and for secondary prevention. Several studies to date have evaluated deep learning methods to detect pulmonary disease by CXR, including evaluations of the efficacy of convolutional neural networks (CNNs) to screen for tuberculosis on CXR^1^ and the construction of a CXR database, called ChestX-ray14, for classification and localization of benchmark lesions^2^. Using these data, long short-term memory (LSTM)^3^ has been applied to the encoded features using a type of DenseNet, allowing the model to exploit dependencies among labels. In addition, a 121-layer CNN utilized to detect pneumonia was found to outperform previous state-of-the-art methods in the further evaluation of 14 diseases^4^. These studies, however, did not include a detailed validation, and concerns regarding the purity of the data entered into the ChestX-ray14 dataset still exist. Moreover, localization results were somewhat unclear and were not sufficient to resolve the suspicion about whether the network includes properly trained and complicated disease patterns. It would be difficult for the CNN model to directly train various sizes and types of complex disease patterns using entire CXR images with weak labels alone. These complex problems may be solved, at least in part, by curriculum learning, which involves gradual training of more complex concepts^5^. Using this strategy, we propose a curriculum for the fine-tuning of complicated whole images after training on lesion-specified patch images. We hypothesized that following patch-based training, this curriculum can guide the network toward better local minima. This study therefore evaluated the efficacy of the curriculum strategy, which trains with two steps, for detecting pulmonary abnormalities on CXR images from two hospitals.

## Results

Test data comprised 20% of the total dataset and included 1423 normal individuals and 1549 patients, including 394 with nodules, 282 with consolidation, 286 with interstitial opacity, 465 with pleural effusion, and 253 with pneumothorax. Considering the healthy subjects, serious data imbalance problems could occur and confuse accurate evaluation of the presence or absence of each disease. Therefore, six performance metrics were calculated separately for each disease class: area under the curve (AUC), accuracy, sensitivity, specificity, positive predictive value (PPV), and negative predictive value (NPV). In addition, the performance of the model in terms of abnormal screening was evaluated by combining the disease classes.

### Comparison with baseline model

**Figure 1:**
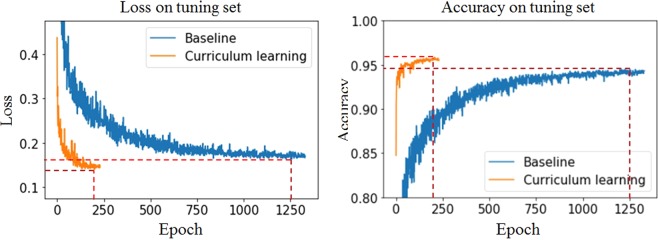
Training curves of a curriculum learning-based model and a baseline model on the tuning dataset.

To assess the effectiveness of the curriculum strategy, a curriculum learning-based model was compared with a baseline model that was trained directly using entire images. Both models were found to be sufficiently trained and to converge on the validation set (Fig. 1). Because the curriculum learning-based model preceded training on the patch images, the convergence was rapid and stable and attained better local minima. Furthermore, the loss and accuracy of the Asan Medical Center (AMC) dataset were 0.116 and 96.1% in baseline and 0.099 and 97.2% in curriculum learning, respectively. The loss and accuracy of the Seoul National University Bundang Hospital (SNUBH) dataset were 0.210 and 92.7% in baseline and 0.181 and 94.2% in curriculum learning, respectively, as shown in Table 1.

**Table 1:** Results of the curriculum learning-based and baseline models at the minimum loss point.

		Baseline	Curriculum learning
Epoch		1257	**199**
Loss	AMC	0.116	**0.099**
	SNUBH	0.210	**0.181**
Accuracy (%)	AMC	96.1	**97.2**
	SNUBH	92.7	**94.2**

Abbreviations: AMC, Asan Medical Center; SNUBH, Seoul National University Bundang Hospital.

As shown in Table 2, the performances of the curriculum learning-based model showed better overall results. The AUCs of abnormalities in the two centres on the curriculum learning-based model were 99.0% and 100.0%, respectively, and those on the baseline model were 96.7% and 99.9%, respectively. The disease patterns with the most difference between the two algorithms in terms of the AUC in the datasets of the two centres were nodule[s] and consolidation. Furthermore, we conducted the paired t-test between the curriculum learning-based model and baseline model. These results show that the performance of the curriculum learning-based model was significantly better in Table 2.

**Table 2:** Classification of subjects in the test dataset using the curriculum learning-based and baseline models.

		AMC						SNUBH					
		AUC	Acc	Sen	Spe	PPV	NPV	AUC	Acc	Sen	Spe	PPV	NPV
Nodule	Baseline	86.7	83.8	83.9	**83.5**	**97.9**	35.7	79.3	70.6	69.1.	**77.4**.	**92.6**	37.7
	Curriculum learning	**92.8**	**96.1**	**98.2**	75.8	97.4	**82.1**	**82.7**	**85.3**	**89.9**	66.5	91.7	**61.6**
Consolidation	Baseline	87.3	90.8	92.2	**68.9**	**97.9**	37.4	84.5	77.7	77.2	**80.4**	**95.7**	38.4
	Curriculum learning	**89.3**	**95.3**	**98.1**	54.6	97.0	**65.7**	**91.2**	**89.7**	**94.5**	62.6	93.5	**66.7**
Interstitial opacity	Baseline	97.5	98.1	98.4	**88.5**	**99.7**	60.5	98.9	96.1	96.5	94.4	98.4	88.0
	Curriculum learning	**98.2**	**98.7**	**99.2**	80.8	99.5	**75.0**	**99.8**	**97.7**	**98.0**	**96.6**	**99.0**	**93.0**
Pleural effusion	Baseline	99.1	97.6	98.2	94.4	99.0	89.8	97.7	93.1	92.6	95.4	98.9	73.9
	Curriculum learning	**99.3**	**98.1**	**98.6**	**94.8**	**99.1**	**92.1**	**98.9**	**95.3**	**95.1**	**96.4**	**99.2**	**81.1**
Pneumothorax	Baseline	94.8	97.2	97.9	80.0	99.3	57.8	97.1	95.6	96.4	91.4	98.2	84.3
	Curriculum learning	**95.3**	**99.3**	**99.9**	**81.5**	**99.3**	**96.4**	**99.1**	**98.3**	**99.2**	**94.1**	**98.8**	**96.2**
Abnormalities	Baseline	96.7	92.2	80.2	98.8	97.5	90.2	99.9	98.2	**98.4**	99.5	99.9	**92.8**
	Curriculum learning	**99.0**	**94.7**	**85.4**	**99.8**	**99.7**	**92.5**	**100.0**	**98.3**	97.9	**100.0**	**100.0**	92.0

^*^Paired t-test; overall abnormalities: p < 2.667e-10, nodule[s]: p < 5.705e-05, consolidation: p < 0.002456, interstitial opacity: p < 0.01225, pleural effusion: p < 0.04229, and pneumothorax: p < 0.01329.

Abbreviations: AMC, Asan Medical Center; SNUBH, Seoul National University Bundang Hospital.

### Performance evaluation

The performance of the curriculum learning-based model on the test set was evaluated using weight parameters at the minimum loss point on the validation set. The six metrics were determined separately for the datasets from each of the hospitals (Table 2). The sensitivity and accuracy of pneumothorax was the highest in the datasets of the two centres with sensitivities of 99.9% and 99.2%, respectively, and with accuracies of 99.3% and 98.3%, respectively. The specificity and AUC of pleural effusion were the highest in the AMC dataset (94.8% and 99.3%), with interstitial opacity having the highest specificity and AUC in the SNUBH dataset (96.6% and 99.8%). The sensitivity and AUC of consolidation were 98.1% and 89.3%, the lowest in the AMC dataset, and those of the nodule[s] were 89.9% and 82.7% in the SNUBH dataset, respectively. Consolidation had the lowest specificity of 54.6% and 62.6% in the datasets of the two centres, respectively. Additionally, pneumothorax had the highest PPV (99.3%) in AMC and NPV (96.2%) in the SNUBH dataset.

In addition, we performed statistical analyses on the size of the nodule[s]. The size of the nodule was divided into three categories—3 cm or less, 3–5 cm, and 5 cm or more. As shown in Table 3, the proposed curriculum learning-based method showed better performance than the baseline model for each nodule size. Nodule[s] that were 3 cm or less in size in the AMC dataset showed the best AUC, and the AUC of nodule[s] whose size was over 5 cm in the SNUBH dataset was the best. The accuracies of the three size categories were 90.2%, 91.9%, and 90.5% in the AMC dataset, respectively, and 74.7%, 77.9%, and 79.65% in the SNUBH dataset, respectively.

**Table 3:** Classification of pulmonary nodules per nodule size in the test dataset using the curriculum learning-based and baseline models.

		AMC						SNUBH					
		AUC	Acc	Sen	Spe	PPV	NPV	AUC	Acc	Sen	Spe	PPV	NPV
Nodule (<3 cm)	Baseline	81.1	78.4	78.4	82.8	99.7	5.6	53.3	59.7	60.1	46.4	97.7	2.9
	Curriculum learning	**89.9**	**90.2**	**90.2**	**89.7**	**99.8**	**12.5**	**64.6**	**74.7**	**75.3**	**53.8**	**98.5**	**5.1**
Nodule (≥3 cm and <5 cm)	Baseline	82.1	80.6	80.4	83.9	**99.0**	17.1	71.2	64.5	63.3	**79.1**	**97.2**	15.6
	Curriculum learning	**87.0**	**91.9**	**92.4**	**81.6**	**99.0**	**34.1**	**71.7**	**77.0**	**77.9**	65.5	96.4	**19.9**
Nodule (≥5 cm)	Baseline	**81.5**	79.8	79.6	**83.3**	**99.2**	12.9	72.8	65.8	64.2	**81.4**	**97.1**	19.1
	Curriculum learning	81.2	**90.5**	**91.2**	71.2	98.9	**22.6**	**77.3**	**79.2**	**79.6**	75.0	96.9	**27.2**

**Figure 2:**
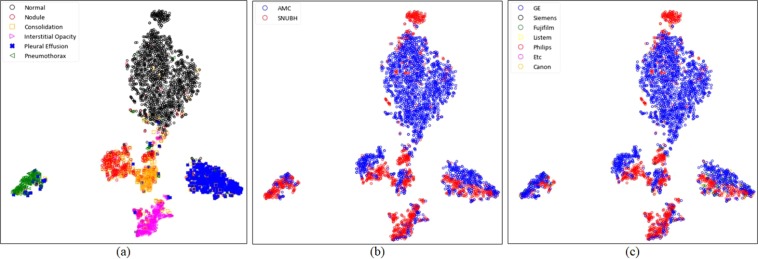
Visualization of manifolds using t-distributed stochastic neighbour embedding (t-SNE), (**a**) manifolds for normal individuals and five types of image patterns of the diseases; (**b**) manifolds for each hospital; (**c**) manifolds according to the manufacturer. (Abbreviations: AMC, Asan Medical Center; SNUBH, Seoul National University Bundang Hospital).

For more detailed evaluation, the variables embedded in the highest layer of the network were visually evaluated after two-dimensional reduction using the t-distributed stochastic neighbour embedding (t-SNE) technique^6^. Figure 2 shows the formation of manifolds according to the disease, the hospital, and the manufacturer of the X-ray machine. The formation of manifolds for each disease and hospital was visualized to assess whether the model correctly trained each disease pattern (Fig. 2). The manifold for each disease was found to be well formed (Fig. 2a). In contrast, the manifolds according to the hospital were found to have a slightly different marginal distribution, as shown in (Fig. 2b). To the best of our knowledge, this phenomenon could be caused by various factors such as the manufacturer of the X-ray machine, radiation exposure time, and image reconstruction algorithm. The most prominent of these factors was the manufacturer, and the formation of the manifold according to the manufacturer was confirmed, as shown in (Fig. 2c). The AMC and SNUBH datasets consisted primarily of data from GE and Philips, respectively, which allowed us to identify different marginal distributions according to the manufacturers.

### Localization using class activation maps

**Figure 3:**
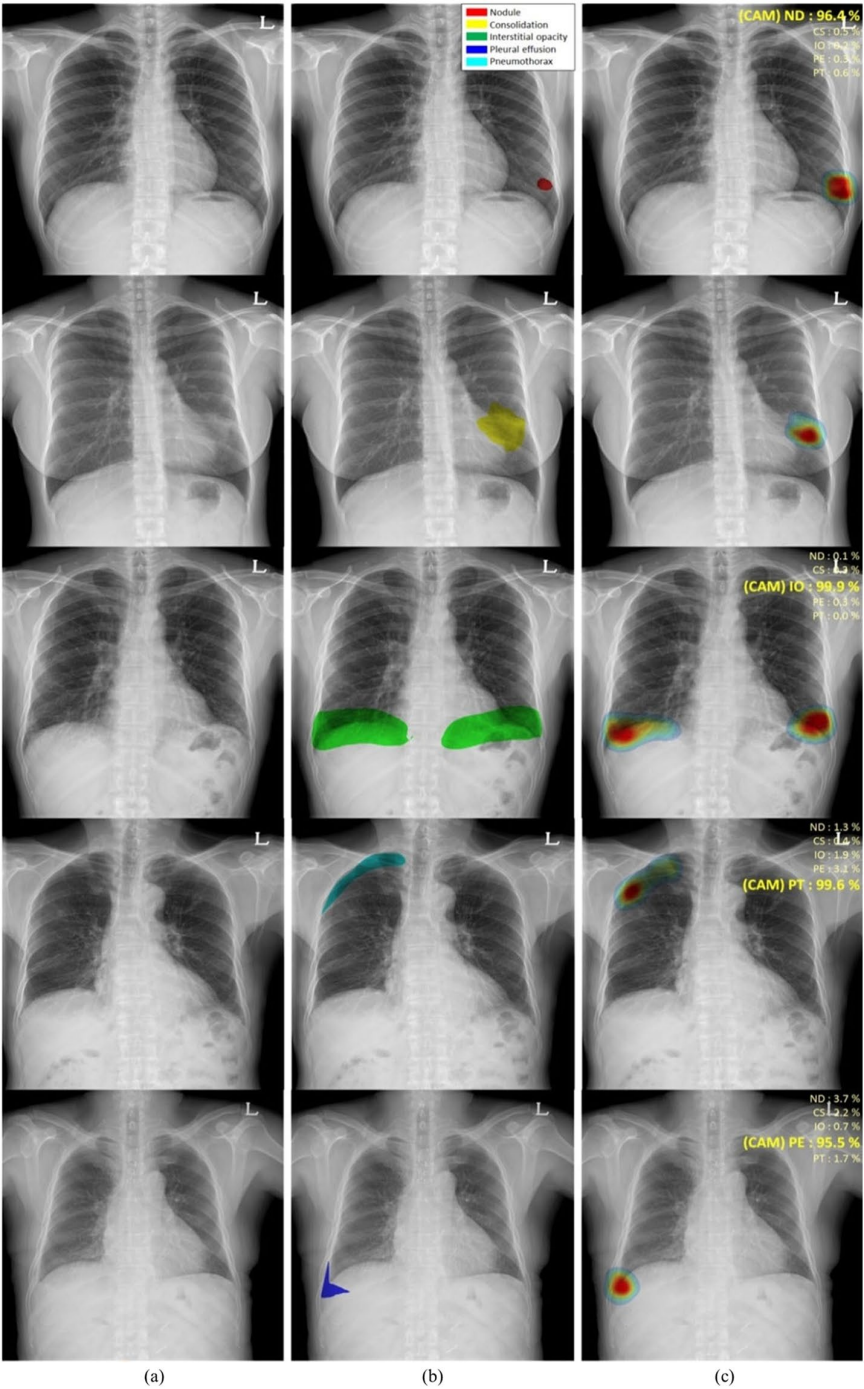
Visualization of the class activation map (CAM) for five pulmonary diseases, (**a**) original images, (**b**) annotations by experts for each disease, and (**c**) visualization by CAM. (Abbreviations: NM, normal; ND, nodule; CS, consolidation; IO, interstitial opacity; PLE, pleural effusion; PN, pneumothorax; CAM, class activation map).

**Figure 4:**
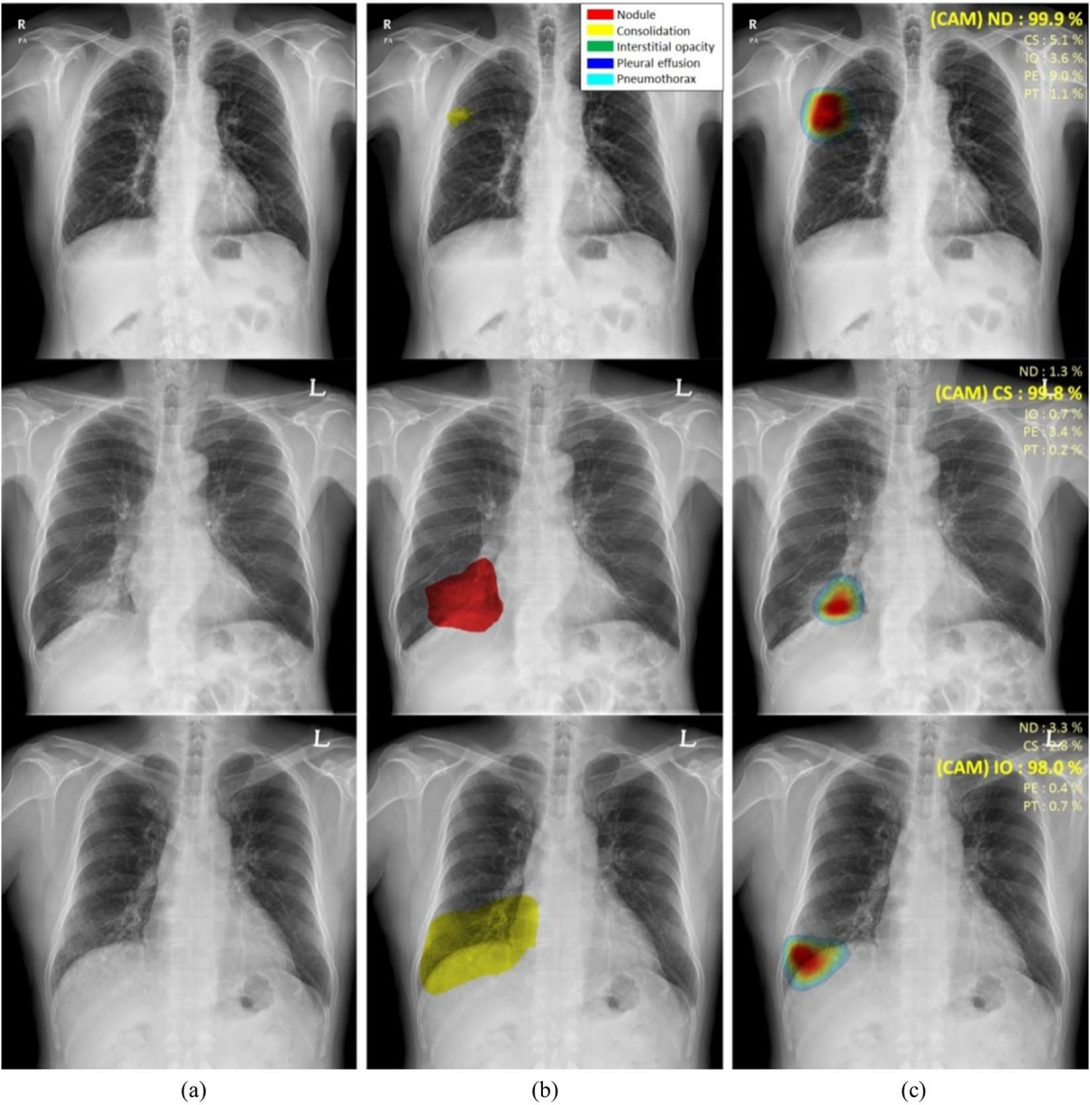
Examples of misclassified nodules and consolidations, (**a**) original images, (**b**) annotations by experts, and (**c**) visualization by CAM. (Abbreviations: NM, normal; ND, nodule; CS, consolidation; IO, interstitial opacity; PLE, pleural effusion; PN, pneumothorax; CAM, class activation map).

**Figure 5:**
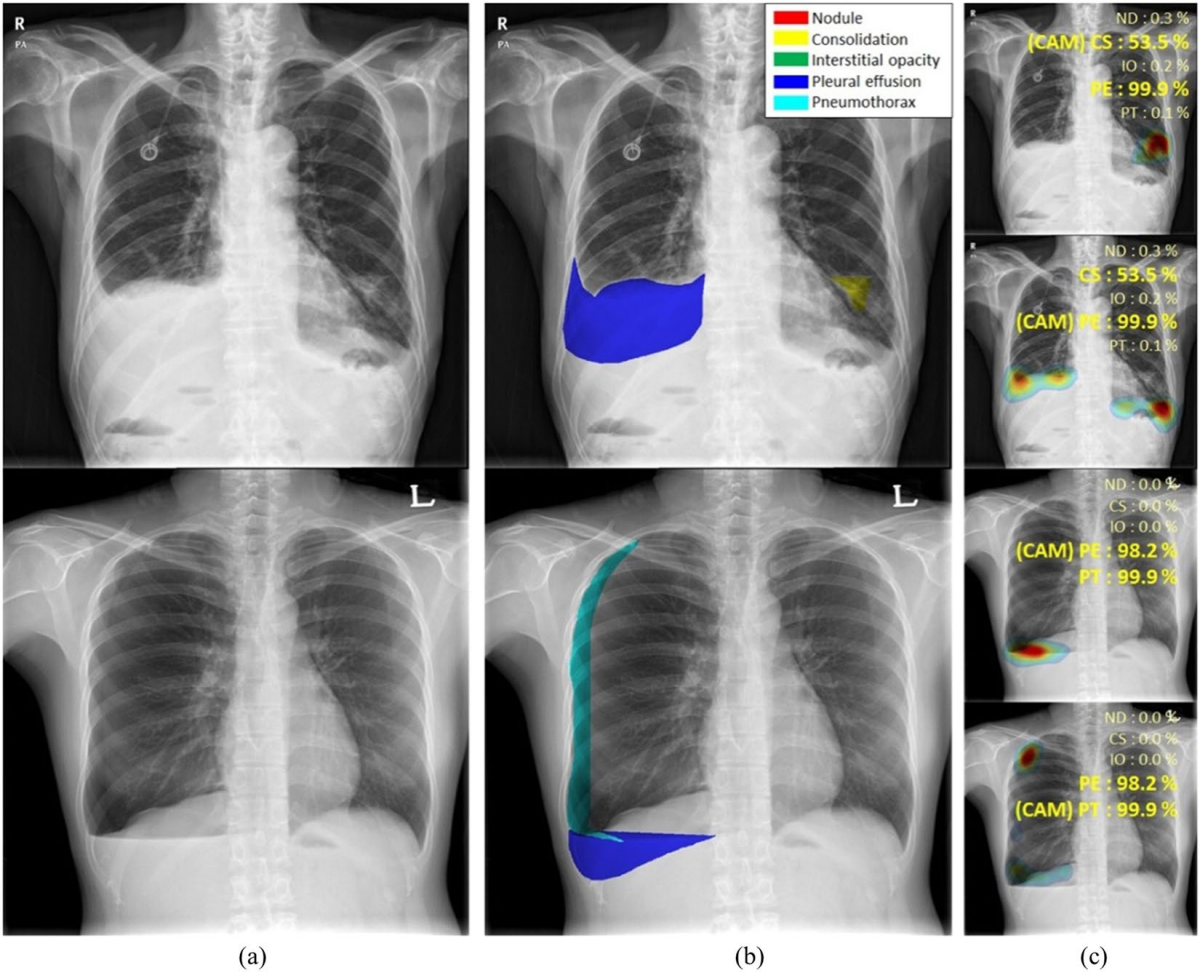
CAMs for patients with multiple diseases, (**a**) original images, (**b**) annotations by experts, and (**c**) visualization by CAM. (Abbreviations: NM, normal; ND, nodule; CS, consolidation; IO, interstitial opacity; PLE, pleural effusion; PN, pneumothorax; CAM, class activation map).

Figure 3 shows the classification results and localization using class activation maps (CAM) for five lung diseases subjected to curriculum learning. Although the CAM results generally coincided with the AUC results drawn by thoracic experts, our CAM algorithms yielded misclassifications in several patients with nodule[s] or consolidation (Fig. 4). Although localization of the disease pattern was visually appropriate, some nodules were classified as consolidations and some consolidations as nodule[s]. Figure 5 also shows the results for patients with multiple diseases. The results of CAM for all trained classes were independently extracted, with the CAMs for the inferred diseases being individually extracted and consistent with the region of interest drawn by the expert.

## Discussion

In this study, we evaluated the efficacy of a two-step curriculum strategy for detecting pulmonary abnormalities on CXR images from two hospitals and found that curriculum learning could guide the model toward a better local minimum. The curriculum learning-based model was investigated using the minimum loss point on the validation set to assess weight parameters on the test set. In general, large disease patterns including interstitial opacity, pleural effusion and pneumothorax showed better performances in this study. Especially, pneumothorax had the highest sensitivity and accuracy in the datasets of the two centres. Furthermore, PPV and NPV of pneumothorax were the best. The specificity of pleural effusion was the highest in the AMC dataset, whereas the specificity of interstitial opacity was the highest in the SNUBH dataset. The AUC of pleural effusion was the highest among other disease patterns. Otherwise, the sensitivity of consolidations was the lowest in the AMC dataset, whereas the specificity of nodule[s] was the lowest in the SNUBH dataset. In addition, the specificity of consolidation was the lowest in the datasets of the two centres and the lowest accuracy in the AMC dataset. Nodule[s] had the lowest accuracy in the SNUBH dataset. Additionally, PPV and NPV results showed better results in the other three lesions than both nodule[s] and consolidation. As shown in Table 2, the curriculum learning-based model showed significantly better overall results. Specifically, the AUCs of abnormalities in the datasets of the two centres on the curriculum learning-based model showed better overall results. Nodule[s] and consolidation showed the most differences between the two algorithms in terms of AUC.

In addition, we conducted various statistical analyses on the size of the nodules. As shown in Table 3, the curriculum learning-based model surpassed the baseline. In our strategy, nodule[s] over 5 cm had the worst AUC in AMC. In addition, as the specificity showed the lowest results, the AMC dataset seems to be confused with consolidation because of the mass type contained in nodule[s]. In contrast, AUC of nodule[s] over 5 cm in the SNUBH dataset was the best. As the nodule[s] size increased, the AUC and accuracy improved. However, overall, the accuracy of nodule[s] was lower than the other disease patterns. Evaluations of the location and classification of nodule[s] and consolidation found that, although CAMs of the disease patterns were appropriately visualised, the two lesions may be misclassified as each other. This may result from similar image patterns with different sizes of nodule[s] and consolidation on CXR images. Differentiating between nodule[s] and consolidation may be difficult in CXRs, suggesting the need for merged labelling criteria.

Although previous studies^7^ have evaluated the classification of each type of lesion, they were unable to accurately determine the locations of various lesions on CXR images during initial diagnosis. The present study included weakly supervised deep learning with curriculum methods that can simultaneously detect multi-labelled lesions of various sizes and disease patterns on large CXR images. Finally, CAM^8^ was used to localise and visualise abnormal patterns. Knowing whether an evaluation of multiple lesions on CXR images by this method yields results like those diagnosed by experts is important. This curriculum-based, weakly supervised strategy may be promising in patients with different types of lesions because it may be used to determine the location and classification of multiple classes of lesions in multi-centre datasets. This result is shown in Fig. 5(a–c). This study had several limitations. Although we have developed a robust model by collecting and training data from datasets of two centres, data need to be collected and evaluated from more hospitals to cover a large number of X-ray variations. Figure 4 shows that untrained X-ray variations would have different marginal distributions and additional generalization methods could be required. Additionally, only five types of lesions were evaluated, suggesting a need to assess the practical utility of this strategy in patients with additional diseases, including cardiomegaly, tuberculosis, rib fracture, and mediastinal widening. In conclusion, this deep learning-based CAD system with high-scale CXR images from two centres, which was evaluated by curriculum learning and weak labelling only, required less preparation to train the system. This system could therefore be easily extended to include various kinds of diseases in actual clinical environments. In addition, our algorithm performed well for the simultaneous detection and classification of five disease patterns—nodule[s], consolidation, interstitial opacity, pleural effusion, and pneumothorax—on CXR images.

## Methods

**Figure 6:**
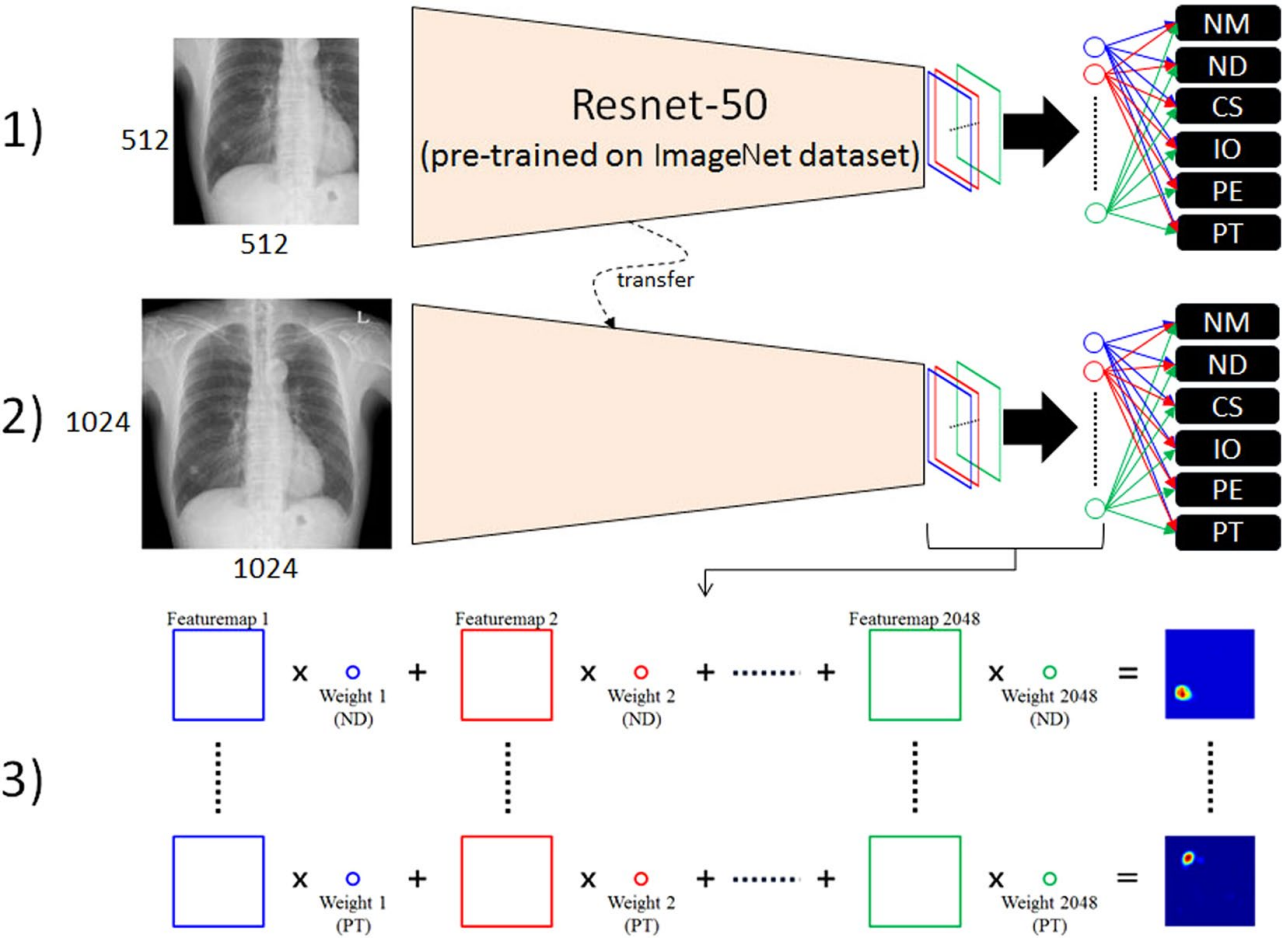
Schematic diagram of the curriculum learning strategy, (1) training patch images, (2) fine-tuning with entire images, and (3) class activation map (CAM). (Abbreviations: NM, normal; ND, nodule; CS, consolidation; IO, interstitial opacity; PLE, pleural effusion; PN, pneumothorax; CAM, class activation map).

The overall procedure for curriculum learning involved two steps. First, the regional patterns of abnormalities were identified by initial learning with patch images, specifically training the network on regional patterns of abnormalities. Second, the network was fine-tuned using entire images. Resnet-50 architecture^9^ was selected to train weak supervisions and modified for multi-label, non-multiclass problems to detect various disease patterns. Finally, class activation maps (CAMs)^8^ were extracted to localise and visualise the abnormal patterns. Figure 6 shows a schematic of the overall procedure.

### Dataset

CXR images of adults were collected from two hospitals, AMC and SNUBH. CXR images of 6069 healthy subjects and 3417 patients at AMC were obtained, with the latter including 944, 550, 280, 1364, and 331 patients with nodule[s], consolidation, interstitial opacity, pleural effusion, and pneumothorax, respectively. CXR images of 1035 healthy subjects and 4404 patients were obtained at SNUBH, with the latter including 1189, 853, 1009, 998, and 944 patients with nodule[s], consolidation, interstitial opacity, pleural effusion, and pneumothorax, respectively. Normal and abnormal datasets with nodule[s] (including mass)/consolidation or interstitial opacities were confirmed by chest CT and pleural effusion, and pneumothorax on CXRs were determined by consensus of two thoracic radiologists with corresponding chest CT images. For training and validation of the model, each abnormal lesion was confirmed and manually drawn by expert thoracic radiologists. The data were randomised, 70% for training, 10% for validation, and 20% for testing. All patient identifiers were removed. The study protocol was approved by the institutional review board for human investigations at AMC and SNUBH, and the requirement for informed consent was waived owing to the retrospective nature of this study.

### Implementation details and training strategy

**Figure 7:**
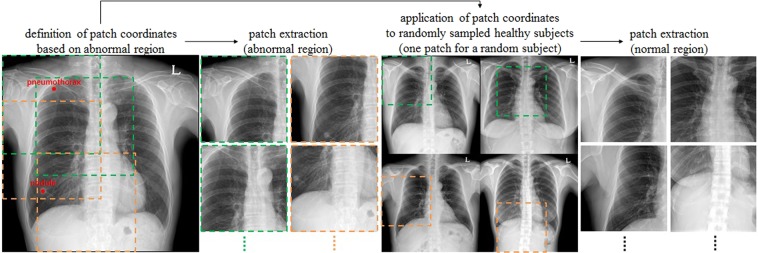
Lesion-based patch extraction for the curriculum learning in chest X-rays (CXRs).

Original CXR images have a high resolution of about 2000 × 2000 pixels. Use of this image size could be problematic because the receptive fields of the model are designed for general natural images, including memory problems. Therefore, all images were converted using bi-linear interpolation to a fixed size of 1024 × 1024 pixels. Because the pixel values of X-ray images have no physical meaning and the noise varies, careful pre-processing was required. The images were adjusted by the average of pixel values for the entire image at that windowing level, and a standard deviation of 3.5 pixel values was used for windowing width, normalizing the range of pixel values for each image. The standard deviation of 3.5 pixel values was selected empirically as a parameter for data augmentation during training by adjusting to a random floating point between 3 and 4. Sample-wise standardization was performed by subtracting the average pixel value of each image. Because patterns on X-ray images may differ among manufacturers, a sharpening and blurring technique was randomly applied to images during training, allowing the model to be robust to these image variations. We used the data in training and included additional data augmentation techniques, such as rotation (±10°), zoom (±10%), and shifting (±10%). The model was implemented in Keras with a Tensorflow backbone and a stochastic gradient descent optimizer with learning and decay rates of 5e-5. In the weakly supervised classification problem, Resnet has been the most widely used CNN architecture because it showed good performance in the ImageNet Large Scale Visual Recognition Competition (ILSVRC). As the layer becomes deeper, high performance could be expected. However, because of the trade-off with training time, this study used a 50-layer architecture (Resnet-50), which showed appropriate training time and performance. Resnet-50 designed in ILSVRC employs a softmax function as a classifier for multiclass problems. By contrast, the detection of various disease patterns on CXR should be regarded as a multi-label problem because various diseases can exist independently^3^. Therefore, the classifier was modified by multiple sigmoid functions:

1 $$\widehat{{y}_{i,j}}=Sigmoid(\widehat{{a}_{i,j}})=\frac{\exp (\widehat{{a}_{i,j}})}{1+\exp (\widehat{{a}_{i,j}})},$$

where $\widehat{{y}_{i,j}}$is the independent probability corresponding to class *j* of sample *i*, and $\widehat{{a}_{i,j}}$is the activation value. Accordingly, loss was calculated as the sum of binary cross-entropy:

2 $${\rm{oss}}(y,\hat{y})={\sum }_{j=0}^{K}{w}_{j}[-\frac{1}{N}{\sum }_{i=0}^{N}\{{y}_{i,j}\cdot \,\log (\widehat{{y}_{i,j}})+(1-{y}_{i,j})\cdot \,\log (1-\widehat{{y}_{i,j}})\}],$$

where *K* and *N* represent the total numbers of classes and samples, respectively, and *w* is a weight term dealing with imbalance. By default, under-sampling was performed to adjust the class distribution of a dataset by randomly sampling the data of the remaining classes based on the class with the smallest number of images during training. Even then, there was a difference of (the number of classes + 1) times considering healthy subjects between the numbers of positive and negative samples in a class. To solve this problem of imbalance, the loss corresponding to each class was multiplied by its weight. Weakly supervised learning for image classification generally requires a considerable amount of data, with more required as complexity increases. Direct training with entire CXR images could lead to the wrong local minima because overlapping patterns of organs, tissues, and bones make the problem more difficult. A simple curriculum learning strategy was employed, consisting of two steps to train the complex disease patterns. In the first step, the Resnet-50 network, which was pre-trained on the ILSVRC dataset^10^, was trained using lesion-specific patch images. These patch images were extracted around the points selected by expert thoracic radiologists to better train the regional patterns of lesions (Fig. 7). The size of each patch image was defined as half its original size to contain a sufficient percentage of each disease pattern, as well as patterns surrounding each lesion. Subsequently, the network was fine-tuned using entire images because of the difference in distribution between patches and entire images.
